# MALDI-TOF MS Based Bacterial Antibiotics Resistance Finger Print for Diabetic Pedopathy

**DOI:** 10.3389/fchem.2021.785848

**Published:** 2022-01-14

**Authors:** Haojie Sun, Peng Lai, Wei Wu, Hao Heng, Shanwen Si, Yan Ye, Jiayi Li, Hehe Lyu, Caiyan Zou, Mengzhe Guo, Yu Wang, Houfa Geng, Jun Liang

**Affiliations:** ^1^ Medical College, Soochow University, Suzhou, China; ^2^ Xuzhou Central Hospital, Xuzhou Clinical School of Xuzhou Medical University, Xuzhou, China; ^3^ Affiliated Hospital of Xuzhou Medical University, Xuzhou, China; ^4^ Jiangsu Key Laboratory of New Drug Research and Clinical Pharmacy, Xuzhou Medical University, Xuzhou, China

**Keywords:** MALDI TOF, macro-proteomics, drug resistance, diabetic foot, finger print

## Abstract

Diabetes mellitus has become a major global health issue. Currently, the use of antibiotics remains the best foundational strategy in the control of diabetic foot infections. However, the lack of accurate identification of pathogens and the empirical use of antibiotics at early stages of infection represents a non-targeted treatment approach with a poor curative effect that may increase the of bacterial drug resistance. Therefore, the timely identification of drug resistant bacteria is the key to increasing the efficacy of treatments for diabetic foot infections. The traditional identification method is based on bacterial morphology, cell physiology, and biochemistry. Despite the simplicity and low costs associated with this method, it is time-consuming and has limited clinical value, which delays early diagnosis and treatment. In the recent years, MALDI-TOF MS has emerged as a promising new technology in the field of clinical microbial identification. In this study, we developed a strategy for the identification of drug resistance in the diagnosis of diabetic foot infections using a combination of macro-proteomics and MALDI MS analysis. The macro-proteomics result was utilized to determine the differential proteins in the resistance group and the corresponding peptide fragments were used as the finger print in a MALDI MS analysis. This strategy was successfully used in the research of drug resistance in patients with diabetic foot infections and achieved several biomarkers that could be used as a finger print for 4 different drugs, including ceftazidime, piperacillin, levofloxacin, and tetracycline. This method can quickly confirm the drug resistance of clinical diabetic foot infections, which can help aid in the early treatment of patients.

## Introduction

Diabetes mellitus has become a major global health issue affecting approximately 9.3% of the population worldwide, and is expected to increase by 25% by 2030 (Sinclair et al., 2020). Approximately 30% of diabetic patients will develop diabetic pedopathy during their lifetime (Armstrong et al., 2017). Of all the complications of diabetes mellitus, diabetic pedopathy poses the most severe risks, and may result in a shortened life expectancy and a dramatic decline in the quality of life (Lavery et al., 2016). In the past few decades, diabetic pedopathy has ranked 10th among all diseases and is often a huge financial burden to the patients family as well as society as a whole (Lazzarini et al., 2018).

At present, antibiotics have been the foundational strategy used to control diabetic foot infections. Due to the lack of accurate identification of the pathogens, the empirical use of antibiotics at the early stages of infection represents a non-targeted treatment approach with a poor curative effect, and may result in bacterial drug resistance (Munita and Arias, 2016). The World Health Organization has recognized antibiotic resistance as one of the most important public health threats in the 21st century (World Health Organization, 2014). The ineffective use of antibiotics will hinder wound healing in patients with diabetic foot infections, and increase the length of stay in hospitals as well as hospital costs. Therefore, timely identification of bacteria and drug resistance is the key in the appropriate treatment of diabetic foot infections (Caruso et al., 2021).

Enterobacterales and *Staphylococcus aureus* are the most common pathogens identified in diabetic pedopathy (Ramirez et al., 2018; Alexi et al., 2021). They play a crucial role in MDR organisms, and their broad antibiotics resistance has increasingly attracted more attention from healthcare associated workers worldwide. Especially concerning is the resistance to cephalosporin and penicillin antibiotics, which may lead to life-threatening issues in the treatment of diabetes-related infections (Weinstein et al., 2019). Over the last decade, the resistance of Enterobacterales to cephalosporin has dramatically increased worldwide. The underlying resistance mechanisms are complex and difficult to be identify. Additionally, carbapenem-hydrolyzing enzymes produced by bacterium are also of great concern (Mao et al., 2017). For example, Klebsiella pneumonia, which has the characteristics of easy-spreading and extensive-contaminating, could have a detrimental effect in its surroundings, such as healthcare settings (Tascini et al., 2015). *S aureus* has primarily exhibits penicillin-resistance. Due to the horizontal transfer and natural selection of genes that encode for a mutant penicillin-binding protein (Masters et al., 2020), this bacteria has a low affinity to penicillin molecules, which underlies the penicillin-resistance.

Bacterial identification is a crucial component of clinical practice. The traditional method is based on the assessment of bacterial morphology, cell physiology, and biochemistry (Sun et al., 2021). While the simplicity of this method and low costs make it an attractive option, it is time-consuming and has limited clinical value due to its low positive rate (Jasson et al., 2010; Oberhettinger et al., 2020). This results in diagnostic and treatment delays.

In recent years, MALDI-TOF MS has emerged as a promising new technology in the field of clinical microbial identification. It is fast, stable, accurate, sensitive, and has a high resolution (Tan et al., 2012; Scott et al., 2016). Its expanded research application in clinical microorganisms has involved many fields, such as bacterial identification, drug resistance analysis, virulence, and epidemiology (Kostrzewa, 2018). Its potential for the rapid detection of bacterial drug resistance has made it especially attractive to many scholars. It falls within the mass range by giving the protein that is resistant to bacteria, and then analyzes the drug resistance of bacteria by looking for characteristic spectral peaks. This study explores its use in detecting bacterial drug resistance in diabetic foot infections and provides a basis for rapid clinical antibiotic selection.

**FIGURE 1:**
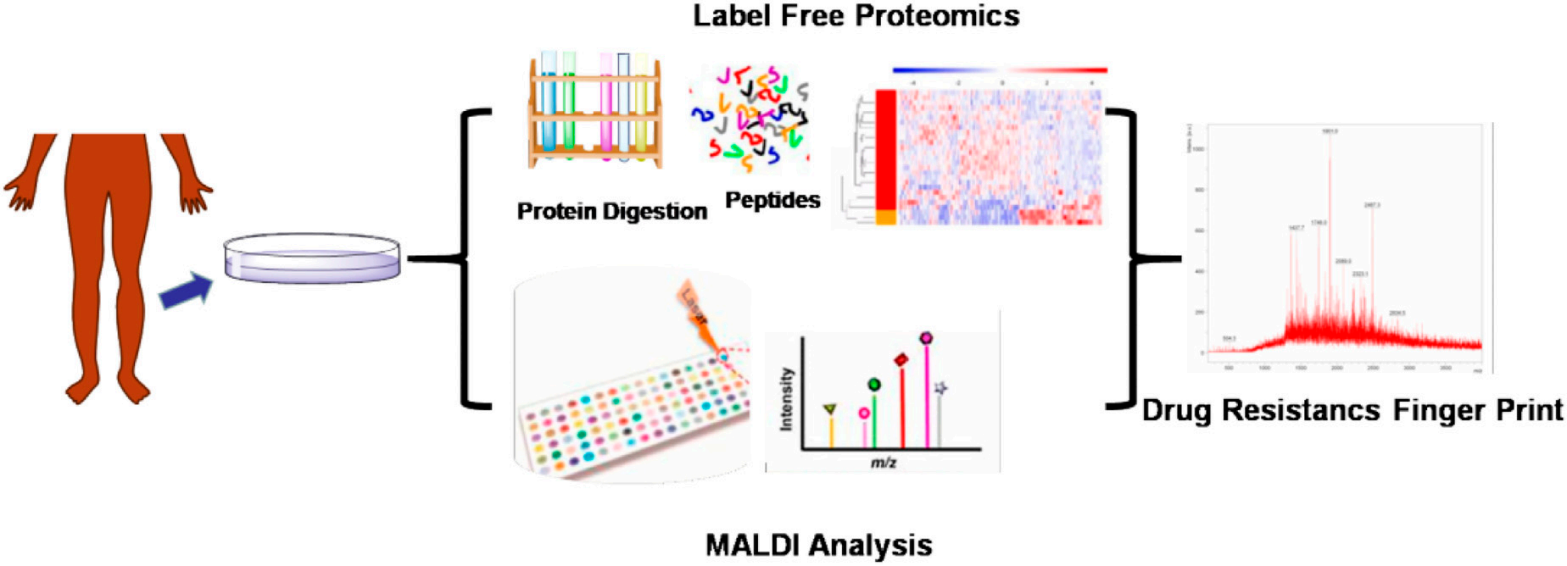
The strategy of the drug resistance finger print research for diabetic foot.

We have developed a MALDI mass spectrometry (MS) finger-print analysis to be used in the diagnosis of antibiotic resistance in patients with diabetic foot infections (Figure 1). First, the bacteria from the affected tissue of patients were cultured and the species were identified. The bacterial cultures were then tested for drug resistance and divided into either a drug-resistant or drug-sensitive group. Next, the bacterial macro-proteomics analysis was introduced to identify the differential proteins between these two groups. Finally, the fragments of the identified differential proteins were compared using the MALDI analysis to identify the drug-resistance finger print. This study could aid in the establishment of a rapid drug resistance MALDI identification method to be used for the clinical the determination of treatments for diabetic foot infections.

## Experimental

### Patient Characteristics

The subjects of the study included type 2 diabetic foot patients hospitalized in the Department of Endocrine and Metabolic Diseases of the Xuzhou Central Hospital from June 2021 to August 2021. The main inclusion criteria were: age was within the range of 18–80 years old, and Wagner 3–4. Exclusion criteria: no standing debridement, immunosuppressants had been used within 3 months prior to admission, and the use of antibiotics in the 4 weeks prior to admission. The diagnosis of diabetes mellitus is based on the 1999 WHO diabetes mellitus diagnostic criteria. The diagnosis of diabetic foot is based on the International Diabetes Foot working group (IWGDF) guide (Lipsky et al., 2020). This study was designed and implemented according to the declaration of Helsinki. All patients and their families were informed and agreed to participate in the study, which was approved by the hospital ethics committee of Xuzhou central hospital.

### Specimen Collection

After flushing the wound with sterile saline, a well-trained diabetic podiatrist took the deep necrotic tissue of the wound to avoid superficial tissue contamination, which could cause inaccurate results. A total of 16 necrotic tissue specimens were collected and divided into two parts under sterile conditions: one part was used for conventional microbial culture and the other was used for MALDI TOF MS analysis.

### Bacterial Inactivation and Protein Extraction

Bacterial inactivation was performed before MS analysis. The bacterial colonies after 72 h incubation were resuspended in 200 μl of pure water. The concentration of bacteria was chosen to be 5 × 10^6^ CFU/ml. Then 600 μl of methanol (MeOH) was added to obtain a final 75% MeOH. This mixture was incubated for 15 min and then centrifuged at 12,000 rom for 10 min to obtain a bacterial pellet. The supernatant was removed and the bacterial pellet was resuspended in 100% acetonitrile (ACN) with 0.1% formic acid. The mixture was passed through the secondary centrifugation (also 12,000 rpm for 10 min) and the bacterial proteins were extracted.

### MALDI TOF MS Method

MALDI TOF MS analysis was performed by a Bruker ultrafle Xtreme MALDI TOF/TOF mass spectrometry (Bruker Daltonics, France). α-cyano-4- hydroxycinnamic acid (HCCA) was used as the matrix and was dissolved into ACN. The extracted proteins were dissolved into water and 1 µl of protein solution was mixed with 1 µl of matrix. The mixture was dripped on a reusable polished steel target and left to dry. The ion mode was positive ion mode with delay: 150 ns; ion source 1 voltage: 20 kV; ion source 2 voltage: 18 kV; lens voltage: 6 kV. All spectra were shown baseline-subtracted, smoothed, and auto-scaled in the *Y*-direction, covering a range of 300–3,000 Da.

### Label Free Macro-Proteomics Analysis

100 µg of protein was reduced with 5 m Mdithiothreitol (DTT) for 1 h at 37°C and subsequently alkylated with 10 mM iodoacetamide for 45 min at RT (room temperature) in the dark. Samples were diluted 1:3 with 50 mM Tris-HCl (pH 8.0) and subjected to proteolytic digestion with trypsin (Promega) at a 1:50 enzyme-to-substrate ratio and incubated overnight at RT. The digested samples were then acidified with 50% trifluoroacetic acid (TFA, Sigma) to a pH value of approximately 2.0. Tryptic peptides were desalted on reversed-phase C18 SPE columns and dried using a Speed-Vac.

Peptides (0.8 μg) were separated on an Easy nLC 1200 UHPLC system (Thermo Scientific) on an in-house packed 20 cm × 75 mm diameter C18 column. The column the flow rate was 0.200 μL/min with 0.1% formic acid and 2% acetonitrilein water (A) and 0.1% formic acid, 90% acetonitrile (B). The peptides were separated with a 6–30% B gradient in 84 min and analyzed using the Thermo Velos mass spectrometer (Thermo Scientific). Parameters were as follows: MS1: resolution—60,000, mass range 350–1800 m/z, MS2: resolution −=50,000, high-energy collision dissociation activation energy (HCD) was 37 eV, isolation width (m/z) was 0.7, AGC Target was 2.0 e5, Max IT was 10^5^ ms.

### Data Statistics

Experiments were repeated at least three times with consistent results. Data are presented as the Mean ± SEM (standard error of the mean) or Mean ± SD (standard deviation). Differences between groups were determined using a two-tailed Mann-Whitney U test or two-tailed Student’s *t*-test. Pearson correlation coefficients (r) were calculated to evaluate correlation and statistical significance was assessed by a two-tailed *t*-test. The results of western blot analysis are the representative images of at least three independent experiments. For boxplots, the center line represents the median, the box limits show the upper and lower quartiles, and the outliers are represented as individual data points.

## Results

**FIGURE 2:**
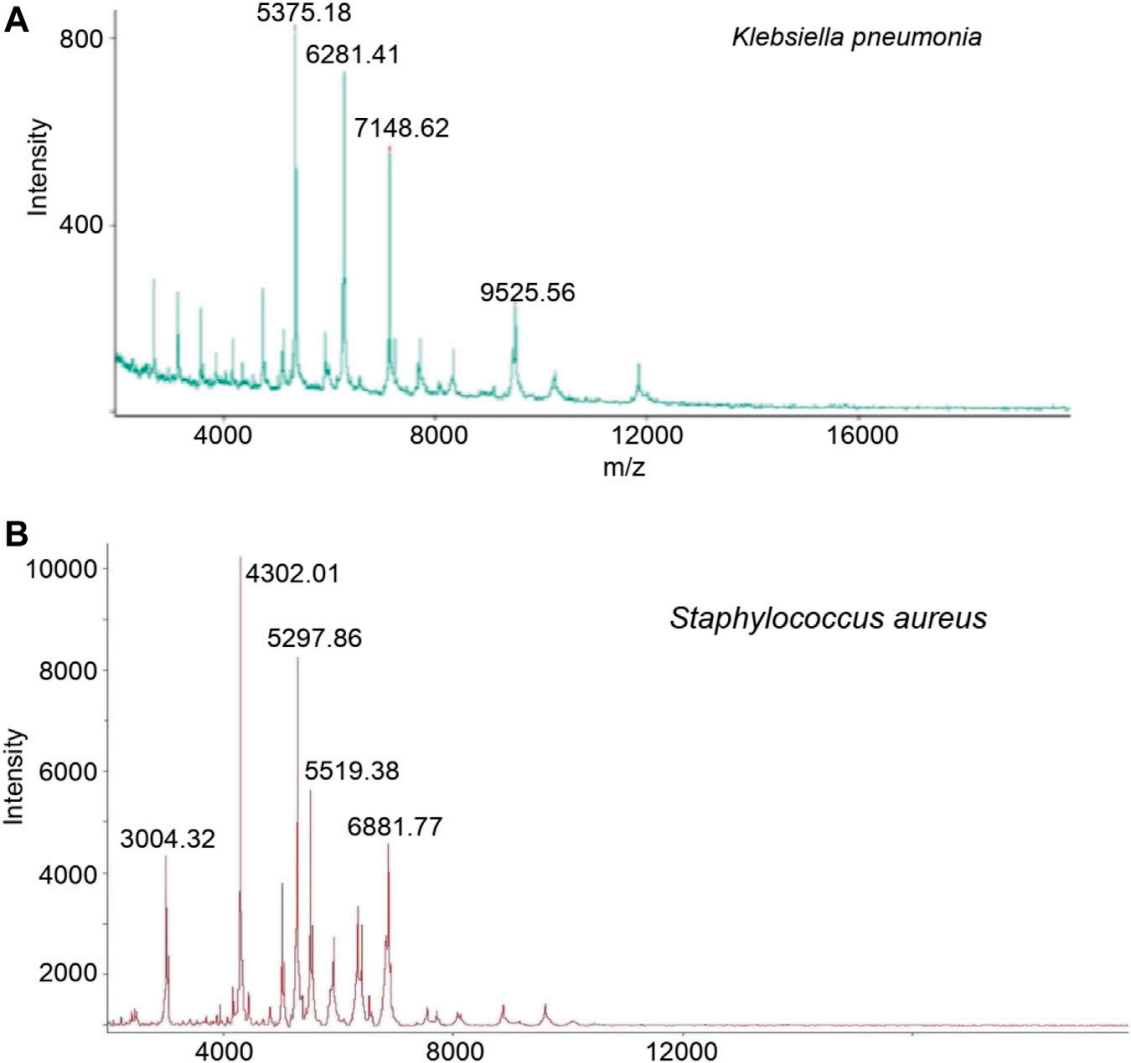
The identification of *Klebsiella pneumonia* **(A)** and *Staphylococcus aureus* **(B)** based on MALDI TOF analysis after bacterial cultured.

A total of 16 samples from patients with diabetic foot infections were collected and used to create bacterial cultures. 5 Gram-positive strains and 11 Gram-negative strains were successfully cultivated. Under the authentication of MALDI MS, the Gram-positive strains consisted of *staphylococcus aureus* and the Gram-negative strains consisted of bacillus (Figure 2). The bacterial cultures were then processed to determine drug-resistance. Both the *staphylococcus aureus* and bacillus were tested using 2 types of drug resistance experiments. In the case for bacillus, ceftazidime and piperacillin were chosen as the test drugs, and levofloxacin and tetracycline were chosen for *staphylococcus aureus*. The proteins in these bacterial cultures were then extracted and passed through the macro-proteomics and MALDI analysis.

**FIGURE 3:**
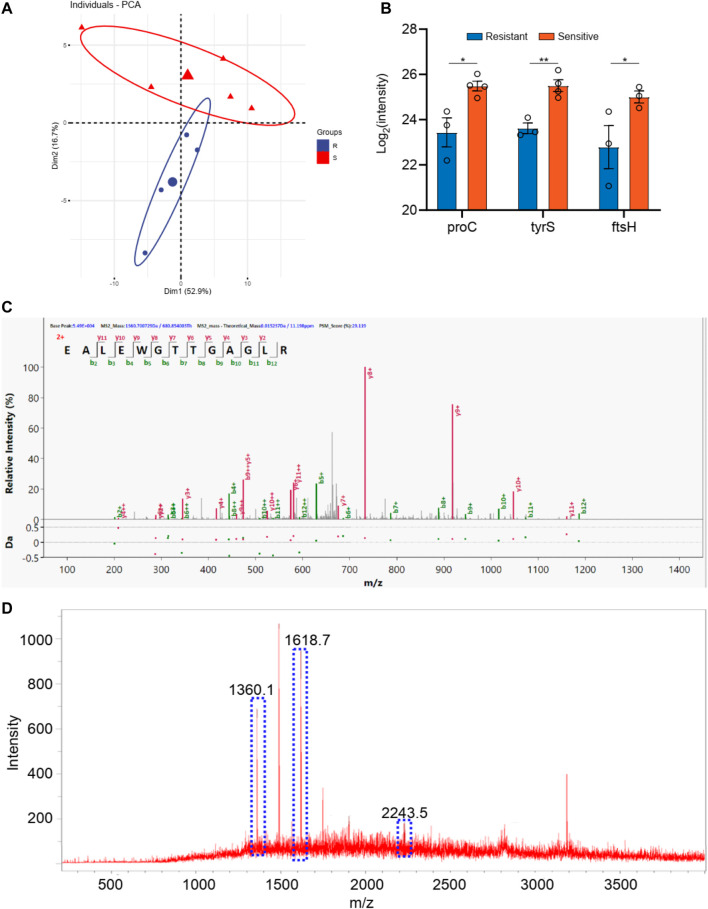
**(A)** The PCA analysis of drug resistance of ceftazidime for *Klebsiella pneumonia*; **(B)** The differential proteins between drug resistance group and drug sensitive group; **(C)** The corresponding differential peptides and **(D)** the finger print in MALDI TOF.

For the bacillus, a total 1,500 proteins were detected by the macro-proteomics analysis. Additionally, there were over 500 identical proteins among them. The samples were then divided into two groups according to the drug resistance test results. As shown in Supplementary Table S1, there are five bacillus samples in the ceftazidime resistance group and six bacillus samples in the other group. Moreover, PCA analyses were carried out for these two groups according to the identical proteins. As shown in Figure 3, these two groups can be well distinguished, with the Q^2^ over 0.8. A total of 10 differential proteins (*p* < 0.05 in *t*-test) were obtained, including fumC, fmt, rpsT, proX, fisH, nuoC, proC, tyrS, ribC, and bfr. Additionally, the digested peptides fragments were found in the database, and were compared with the MALDI TOF analysis. Finally, three peptides were consistently obtained in both results (Figures 3C,D, Supplementary Figure S1), including the peptides from nuoC with a sequence of EALEWGTTGAGLR (m/z 1,360.7), from fisH with a sequence of ESTAYHEAGHAIIGR (m/z 1,611.8), and from tyrS with a sequence of LAEEIIYGPEHVSTGASNDIK (m/z 2243.1). These three peaks of peptides can be considered the finger print for the resistance of bacillus to ceftazidime.

**FIGURE 4:**
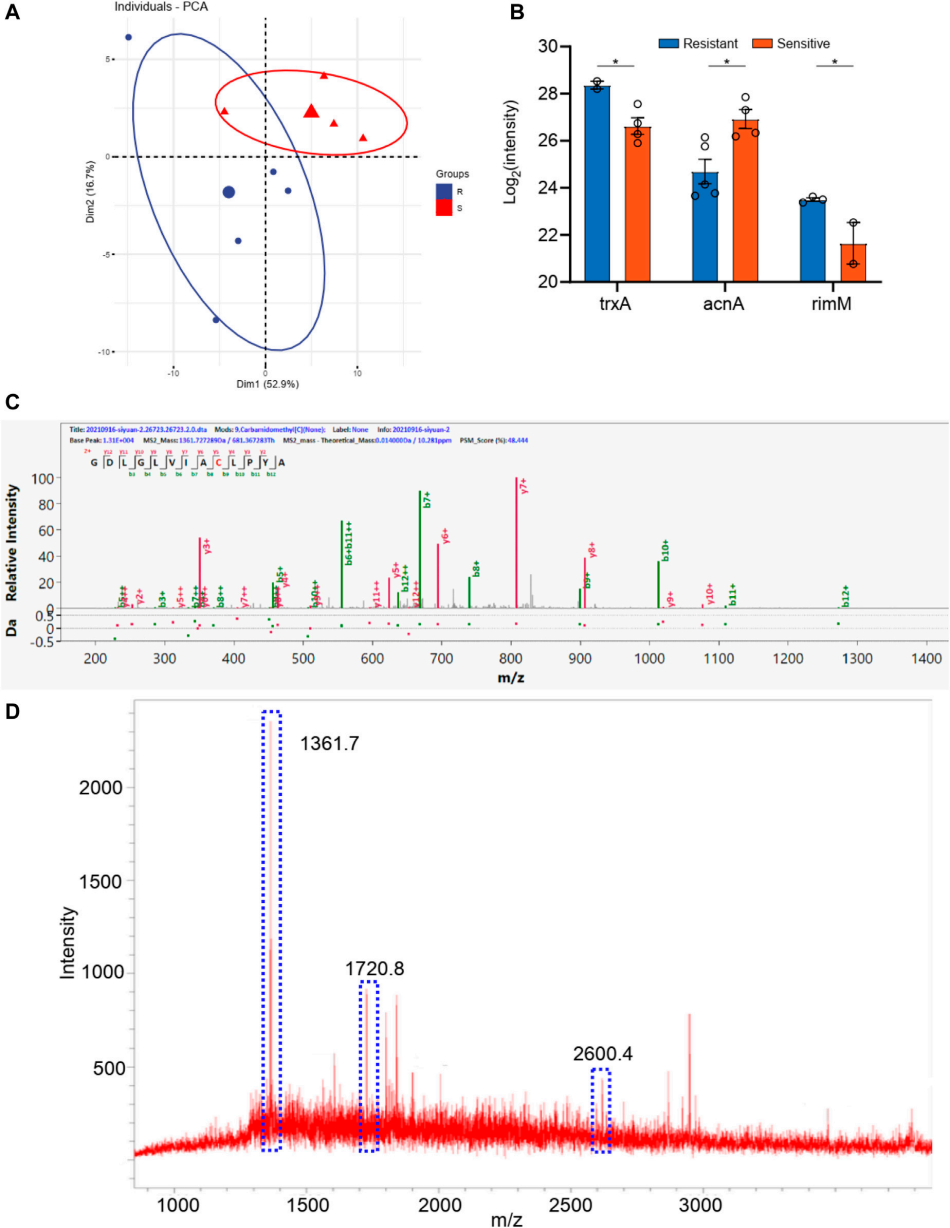
**(A)** The PCA analysis of drug resistance of piperacillin for *Klebsiella pneumonia*; **(B)** The differential proteins between drug resistance group and drug sensitive group; **(C)** The corresponding differential peptides and **(D)** the finger print in MALDI TOF.

The samples were also divided into two groups according to piperacillin resistance. As shown in Supplementary Table S1, there are six bacillus samples in the piperacillin resistance group and five bacillus samples in the other group. The PCA analyses were carried out for these two groups according to the identical proteins. As shown in Figure 4, these two groups can be well distinguished, with the Q^2^ over 0.8. A total of 14 differential proteins (*p* < 0.05 in *t*-test) were obtained, including ybjP, rpmI, rof, rim, acnA, lolA, engB, hisS, bglX, cpxR, phoP, trxA, ompA, and bfr. Additionally, the digested peptides fragments were found in the database, which were compared with the MALDI TOF analysis. Finally, three peptides were obtained consistently in both results (Figures 4C,D, Supplementary Figure S2), including the peptides from acnA with a sequence of SDTYGWQEDSTYIR (m/z 1720.8), from ompA with a sequence of ATLKPEGQAALDQLYSQLSNLDPK (m/z 2600.4), and from rpmI with a sequence of GDLGLVIACLPYA (m/z 1,361.7). These three peaks of peptides can be considered as the finger print for the resistance of bacillus to piperacillin..

**FIGURE 5:**
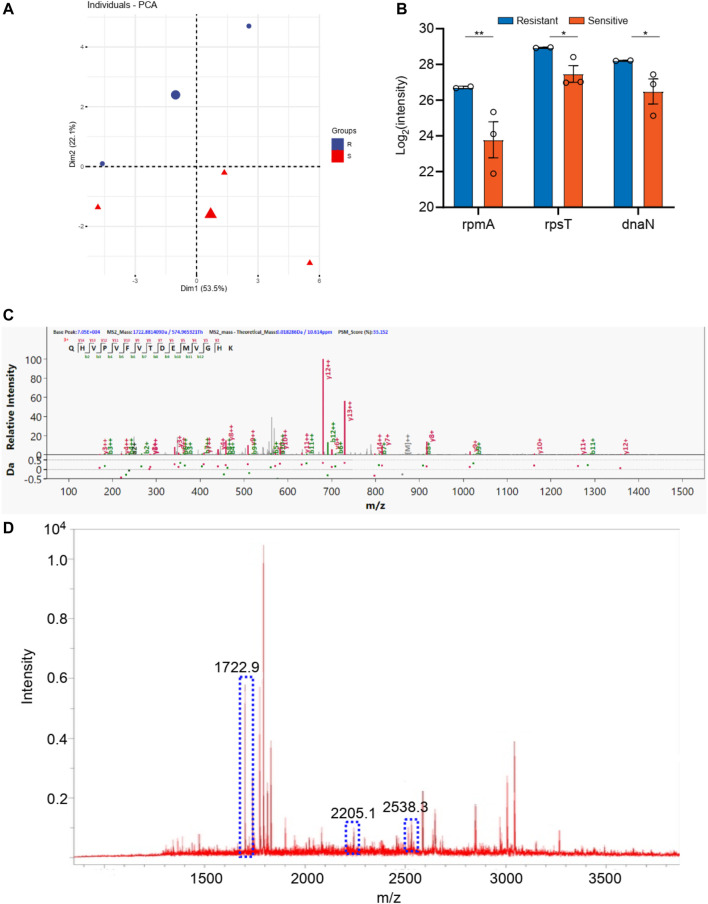
**(A)** The PCA analysis of drug resistance of levofloxacin for *Staphylococcus aureus*; **(B)** The differential proteins between drug resistance group and drug sensitive group; **(C)** The corresponding differential peptides and **(D)** the finger print in MALDI TOF.

For the *staphylococcus aureus*, a total 1,000 proteins were detected by macro-proteomics analysis. In addition, there are over 200 identical proteins among them. The samples were divided into two groups according to the two other drug resistance test results. As shown in Supplementary Table S2, there are two *staphylococcus aureus* samples in levofloxacin resistance group and three *staphylococcus aureus* samples in the sensitive group. Moreover, the PCA analysis were carried out for these two groups according to the identical proteins. As shown in Figure 5, these two groups can be well distinguished with the Q^2^ over 0.8. But only 3 differential proteins (*p* < 0.05 in *t*-test) were obtained, including rpsS, rplV, and rpsQ. Additionally, the digested peptides fragments were found in the database, which were compared with the MALDI TOF analysis. Finally, three peptides were obtained consistently in both results (Figures 5C,D, Supplementary Figure S3), including the peptides from rpsS with the sequence QHVPVFVTDRMVGHK (m/z 1722.9), from rplV with the sequence VLESAIANARHNDGADIDDLKVTK (m/z 2538.3), and from rpsQ with the sequence LHVHDENNECGIGDVVEIR (m/z 2205.1). These three peaks of peptides can be considered as the finger print for the levofloxacin resistance of *staphylococcus aureus*.

**FIGURE 6:**
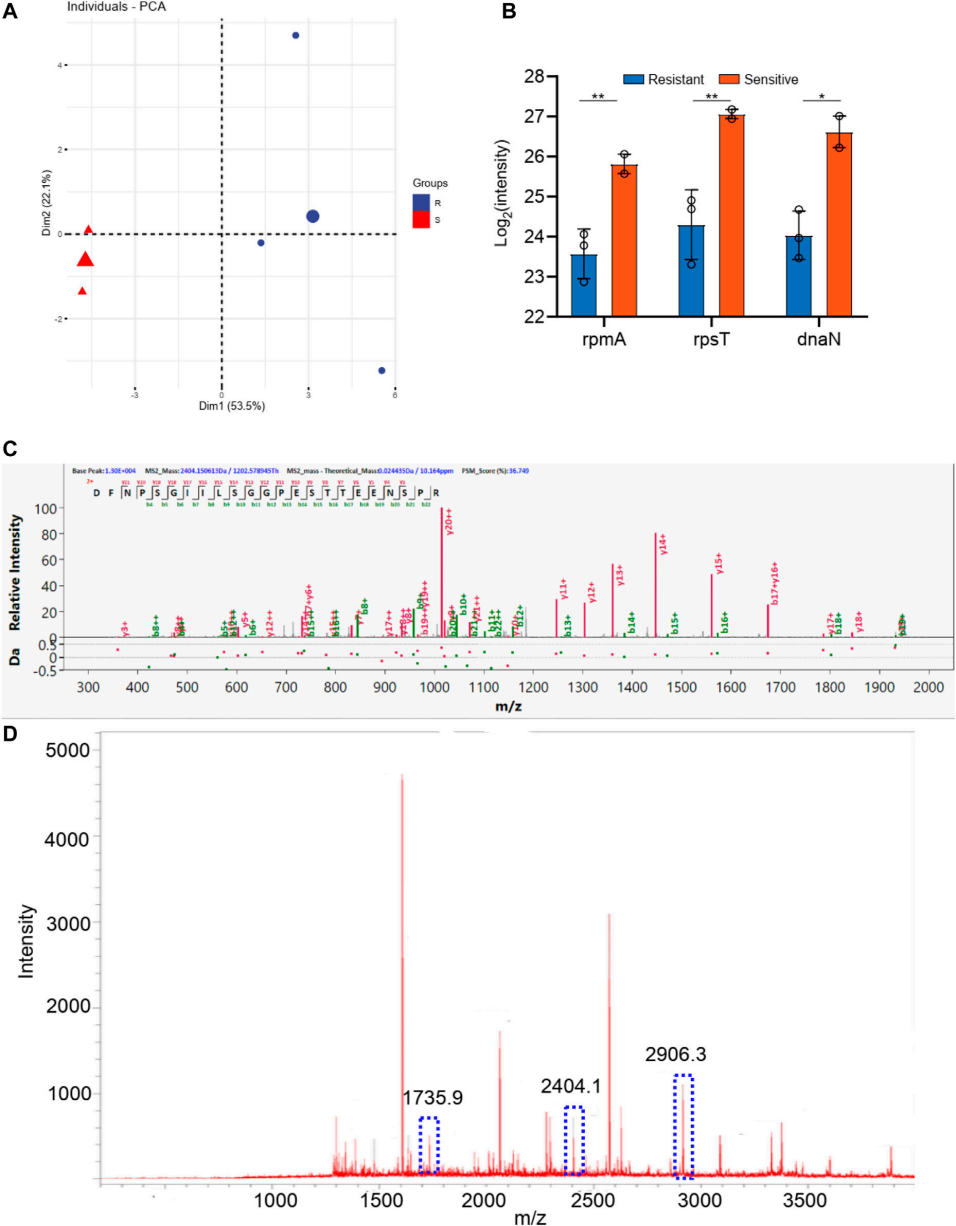
**(A)** The PCA analysis of drug resistance of tetracycline for *Staphylococcus aureus*; **(B)** The differential proteins between drug resistance group and drug sensitive group; **(C)** The corresponding differential peptides and **(D)** the finger print in MALDI TOF.

The *staphylococcus aureus* samples were also divided into two groups according to the tetracycline resistance results. As shown in Supplementary Table S2, there are three *staphylococcus aureus* samples in levofloxacin resistance group and two *staphylococcus aureus* samples in the sensitive group. As shown in Figure 6, these two groups can be well distinguished with the Q^2^ over 0.8. And 10 differential proteins (*p* < 0.05 in *t*-test) were obtained, including rpmA, rpsT, dnaN, guaA, proS, alaS, frr, adk, groS, and rplE. Additionally, the digested peptides fragments were found in the database, which were compared with the MALDI TOF analysis. Finally, three peptides were obtained consistently in both results (Figures 6C,D, Supplementary Figure S4), including the peptides from rplE with the sequence AKLHDYYKDEVVKK (m/z 1735.9), from guaA with the sequence DFNPSGIILSGGPESTTEENSPR (m/z 2404.2), and from proS with the sequence DAYSFHTSQESLQETYDAMYAAYSK (m/z 2906.3). These three peaks of peptides can be considered as the finger print for the tetracycline resistance of *staphylococcus aureus*.

In addition, we added 12 bacterial samples from diabetic podiatry to validate our method, including 7 samples of ceftazidime resistance of bacillus, and 5 samples of levofloxacin resistance of *staphylococcus aureus*. We examined whether there were characteristic peaks of resistance in these samples. It is worth noting that these samples were all tested blind, that is, the tester did not know whether the samples were drug-resistant bacteria samples before the test. The detection results showed that at least one characteristic peak of drug resistance could be detected in these drug resistant bacteria samples, indicating that the method developed by us has good accuracy (Supplementary Table S3, and Supplementary Figure S4, S5).

## Discussion

Recent studies have suggested that the microbiota in diabetic foot infections are diverse, which is typically beyond the identification capabilities of the traditional culture method. Although molecular-based methods, such as qPCR and mNGS, can effectively identify the bacterial species, they are difficult to routinely carry out in clinical practice and also have several inherent biases (Malone et al., 2017; Sadeghpour Heravi et al., 2019). Overall, there is still no simple and specific method to identify the antibiotics resistance of bacteria.

Using bacterial cultures made from diabetic pedopathy patients, we further conducted the MALDI TOF analysis. Consistent with previous studies (Złoch et al., 2021), our study found both Gram-positive and Gram-negative bacterium. A total of 5 strains of Gram-positive bacteria and 11 strains of Gram-negative bacteria were used for further MALDI detection and analysis, which authenticated that the Gram-positive strains consisted of *staphylococcus aureus* and the Gram-negative strains consisted of bacillus. Subsequently, a macro-proteomics analysis was carried out to detect the proteins associated with antibiotic resistance. As a result, a total of 1,500 proteins and 1,000 proteins were detected in the bacillus and *staphylococcus aureus* by macro-proteomics analysis, respectively. Combined with the results of bacterial culture, MALDI TOF, and proteomics, we found a series of differentially expressed proteins. According to the antibiotics resistance, subgroup analyses have confirmed the presence of a finger print of the different bacterium. The peaks of the nuoC, fisH, and tyrS peptides can be considered as the finger print for the resistance of bacillus to ceftazidime resistance. The peaks of the acnA, ompA, and rpmI peptides can be considered as the finger print for the resistance of bacillus to ceftazidime. The peaks of the rpsS, rplV, and rpsQ peptides can be considered as the finger print for the resistance of *staphylococcus aureus* to levofloxacin resistance. The peaks of the rplE, guaA, and proS peptides can be considered as the finger print for the resistance of *staphylococcus aureus* to tetracycline.

An appropriate antibiotic strategy is well known to be essential in the management of diabetic foot infections. However, the prompt identification of antibiotic resistance is the cornerstone of anti-infection therapy. The bacterial resistance fingerprint identified by this study can guide modifications to clinical antibiotic regimens in the earliest period, which may enhance infection control and wound healing of diabetic pedopathy, shorten the length of hospital stays, and reduce overall costs. Based on the convenience and accuracy of the new method in the identification of a bacteria finger print, as well as results from a previous study (Asghari et al., 2021), has confirmed that MALDI TOF could be widely used in clinic and guide decision-making regarding the use of antibiotics.

## Conclusion

In this work, we have developed a strategy for the identification of drug resistance in the diagnosis of diabetic foot. Macro-proteomics and MALDI MS analysis were combined in this strategy. From the macro-proteomics result, the differential proteins which in the resistance group were obtained and the correspondence peptide fragments were used as the finger print in MALDI MS analysis. Then this strategy was successfully used in the drug resistance research in diabetic foot patients and achieved several biomarkers as finger print for 4 drugs, including ceftazidime, piperacillin, levofloxacin, and tetracycline. This method can quickly confirm the drug resistance of diabetic foot in clinical, which can help the treatment of patients as early as possible.

## Data Availability

The original contributions presented in the study are included in the article/Supplementary Material, further inquiries can be directed to the corresponding authors.

## References

[B1] Armstrong D. G. Boulton A. J. M. Bus S. A. (2017). Diabetic Foot Ulcers and Their Recurrence. N. Engl. J. Med. 376 (24), 2367–2375. 10.1056/nejmra1615439 28614678

[B2] Asghari E. Kiel A. Kaltschmidt B. P. Wortmann M. Schmidt N. Hüsgen B. (2021). Identification of Microorganisms from Several Surfaces by MALDI-TOF MS: *P. aeruginosa* Is Leading in Biofilm Formation. Microorganisms 9 (5), 992. 10.3390/microorganisms9050992 34064414 PMC8147854

[B3] Caruso P. Maiorino M. I. Macera M. Signoriello G. Castellano L. Scappaticcio L. (2021). Antibiotic Resistance in Diabetic Foot Infection: How it Changed with COVID-19 Pandemic in a Tertiary Care center. Diabetes Res. Clin. Pract. 175, 108797. 10.1016/j.diabres.2021.108797 33845049 PMC8047299

[B4] Jasson V. Jacxsens L. Luning P. Rajkovic A. Uyttendaele M. (2010). Alternative Microbial Methods: An Overview and Selection Criteria. Food Microbiol. 27 (6), 710–730. 10.1016/j.fm.2010.04.008 20630313

[B5] Kostrzewa M. (2018). Application of the MALDI Biotyper to Clinical Microbiology: Progress and Potential. Expert Rev. Proteomics 15 (3), 193–202. 10.1080/14789450.2018.1438193 29411645

[B6] Lavery L. A. Davis K. E. Berriman S. J. Braun L. Nichols A. Kim P. J. (2016). WHS Guidelines Update: Diabetic Foot Ulcer Treatment Guidelines. Wound Rep. Reg. 24 (1), 112–126. 10.1111/wrr.12391 26663430

[B7] Lazzarini P. A. Pacella R. E. Armstrong D. G. van Netten J. J. (2018). Diabetes-related Lower-Extremity Complications Are a Leading Cause of the Global burden of Disability. Diabet. Med. 35, 1297–1299. 10.1111/dme.13680 29791033

[B8] Lienard A. Hosny M. Jneid J. Schuldiner S. Cellier N. Sotto A. (2021). *Escherichia coli* Isolated from Diabetic Foot Osteomyelitis: Clonal Diversity, Resistance Profile, Virulence Potential, and Genome Adaptation. Microorganisms 9 (2), 380. 10.3390/microorganisms9020380 33668594 PMC7918245

[B9] Lipsky B. A. Senneville É. Abbas Z. G. Aragón‐Sánchez J. Diggle M. Embil J. M. (2020). Guidelines on the Diagnosis and Treatment of Foot Infection in Persons with Diabetes (IWGDF 2019 Update). Diabetes Metab. Res. Rev. 36, e3280. 10.1002/dmrr.3280 32176444

[B10] Malone M. Johani K. Jensen S. O. Gosbell I. B. Dickson H. G. Hu H. (2017). Next Generation DNA Sequencing of Tissues from Infected Diabetic Foot Ulcers. EBioMedicine 21, 142–149. 10.1016/j.ebiom.2017.06.026 28669650 PMC5514496

[B11] Mao W. Xia L. Xie H. (2017). Detection of Carbapenemase-Producing Organisms with a Carbapenem-Based Fluorogenic Probe. Angew. Chem. Int. Ed. 56 (16), 4468–4472. 10.1002/anie.201612495 28332754

[B12] Masters E. A. de Mesy Bentley K. L. Gill A. L. Hao S. P. Galloway C. A. Salminen A. T. (2020). Identification of Penicillin Binding Protein 4 (PBP4) as a Critical Factor for *Staphylococcus aureus* Bone Invasion during Osteomyelitis in Mice. Plos Pathog. 16 (10), e1008988. 10.1371/journal.ppat.1008988 33091079 PMC7608983

[B13] Munita J. M. Arias C. A. (2016). Mechanisms of Antibiotic Resistance. Microbiol. Spectr. 4 (2), 1–24. 10.1128/microbiolspec.VMBF-0016-2015 PMC4888801 27227291

[B14] Oberhettinger P. Zieger J. Autenrieth I. Marschal M. Peter S. (2020). Correction to: Evaluation of Two Rapid Molecular Test Systems to Establish an Algorithm for Fast Identification of Bacterial Pathogens from Positive Blood Cultures. Eur. J. Clin. Microbiol. Infect. Dis. 39 (10), 2003. 10.1007/s10096-020-04012-5 32870443 PMC7645490

[B15] Ramirez H. A. Pastar I. Jozic I. Stojadinovic O. Stone R. C. Ojeh N. (2018). *Staphylococcus aureus* Triggers Induction of miR-15B-5P to Diminish DNA Repair and Deregulate Inflammatory Response in Diabetic Foot Ulcers. J. Invest. Dermatol. 138 (5), 1187–1196. 10.1016/j.jid.2017.11.038 29273315 PMC6358418

[B16] Sadeghpour Heravi F. Zakrzewski M. Vickery K. G. Armstrong D. D. Hu H. (2019). Bacterial Diversity of Diabetic Foot Ulcers: Current Status and Future Prospectives. J. Clin. Med. 8 (11), 1935. 10.3390/jcm8111935 PMC6912738 31717640

[B17] Scott J. S. Sterling S. A. To H. Seals S. R. Jones A. E. (2016). Diagnostic Performance of Matrix-Assisted Laser Desorption Ionisation Time-Of-Flight Mass Spectrometry in Blood Bacterial Infections: a Systematic Review and Meta-Analysis. Infect. Dis. 48 (7), 530–536. 10.3109/23744235.2016.1165350 27118169

[B18] Sinclair A. Saeedi P. Kaundal A. Karuranga S. Malanda B. Williams R. (2020). Diabetes and Global Ageing Among 65-99-Year-Old Adults: Findings from the International Diabetes Federation Diabetes Atlas, 9th Edition. Diabetes Res. Clin. Pract. 162, 108078. 10.1016/j.diabres.2020.108078 32068097

[B19] Sun J. Shi H. Xue Y. Cheng W. Yu M. Ding C. (2021). Releasing Bacteria from Functional Magnetic Beads Is Beneficial to MALDI-TOF MS Based Identification. Talanta 225, 121968. 10.1016/j.talanta.2020.121968 33592721

[B20] Tan K. E. Ellis B. C. Lee R. Stamper P. D. Zhang S. X. Carroll K. C. (2012). Prospective Evaluation of a Matrix-Assisted Laser Desorption Ionization-Time of Flight Mass Spectrometry System in a Hospital Clinical Microbiology Laboratory for Identification of Bacteria and Yeasts: a Bench-By-Bench Study for Assessing the Impact on Time to Identification and Cost-Effectiveness. J. Clin. Microbiol. 50 (10), 3301–3308. 10.1128/JCM.01405-12 22855510 PMC3457442

[B21] Tascini C. Lipsky B. A. Iacopi E. Ripoli A. Sbrana F. Coppelli A. (2015). KPC-producing *Klebsiella pneumoniae* Rectal Colonization Is a Risk Factor for Mortality in Patients with Diabetic Foot Infections. Clin. Microbiol. Infect. 21 (8), e1. 10.1016/j.cmi.2015.04.010 25911991

[B22] Weinstein E. J. Han J. H. Lautenbach E. Nachamkin I. Garrigan C. Bilker W. B. (2019). A Clinical Prediction Tool for Extended-Spectrum Cephalosporin Resistance in Community-Onset Enterobacterales Urinary Tract Infection. Open Forum Infect. Dis. 6 (4), ofz164. 10.1093/ofid/ofz164 31041359 PMC6483753

[B23] World Health Organization (2014). Antimicrobial Resistance: Global Report on Surveillance 2014. Available at: https://apps.who.int/mediacentre/news/releases/2014/amr-report/en/index.html (Accessed September 25, 2021).

[B24] Złoch M. Maślak E. Kupczyk W. Jackowski M. Pomastowski P. Buszewski B. (2021). Culturomics Approach to Identify Diabetic Foot Infection Bacteria. Int. J. Mol. Sci. 22 (17), 9574. 10.3390/ijms22179574 34502482 PMC8431627

