# Supplementary material for: MALDI-TOF MS Based Bacterial Antibiotics Resistance Finger Print for Diabetic Pedopathy
Source: Front Chem. 2022 Jan 14;9:785848. doi: 10.3389/fchem.2021.785848 (PMC8795630; doi:10.3389/fchem.2021.785848)
Supplement: Supplementary file 1 [file DataSheet1.PDF]

Supporting Infromation

for

MALDI-TOF based bacterial antibiotics resistance finger print for diabetic pedopathy

Haojie Sun[1,2†], Peng Lai[2†], Wei Wu[3†], Hao Heng[2], Shanwen Si[2], Yu Wang[2], Caiyan Zou[2], Mengzhe Guo[4], Houfa Geng[2]*, Jun Liang[1,2]*

1 Medical College, Soochow University, Suzhou, 215123, PR, China

2 Xuzhou Central Hospital, Xuzhou Clinical School of Xuzhou Medical University, Affiliated Hospital of Medical School of Southeast University, Xuzhou Clinical School of Nanjing Medical University, Xuzhou Institute of Medical Science, Xuzhou, China

3 Affiliated Hospital of Xuzhou Medical University, Xuzhou, China

4 Jiangsu Key Laboratory of New Drug Research and Clinical Pharmacy, Xuzhou Medical University, Xuzhou, China

Corresponding Author: Jun Liang* : mwlj521@163.com and

Houfa Geng*: genghoufa@njmu.edu.cn

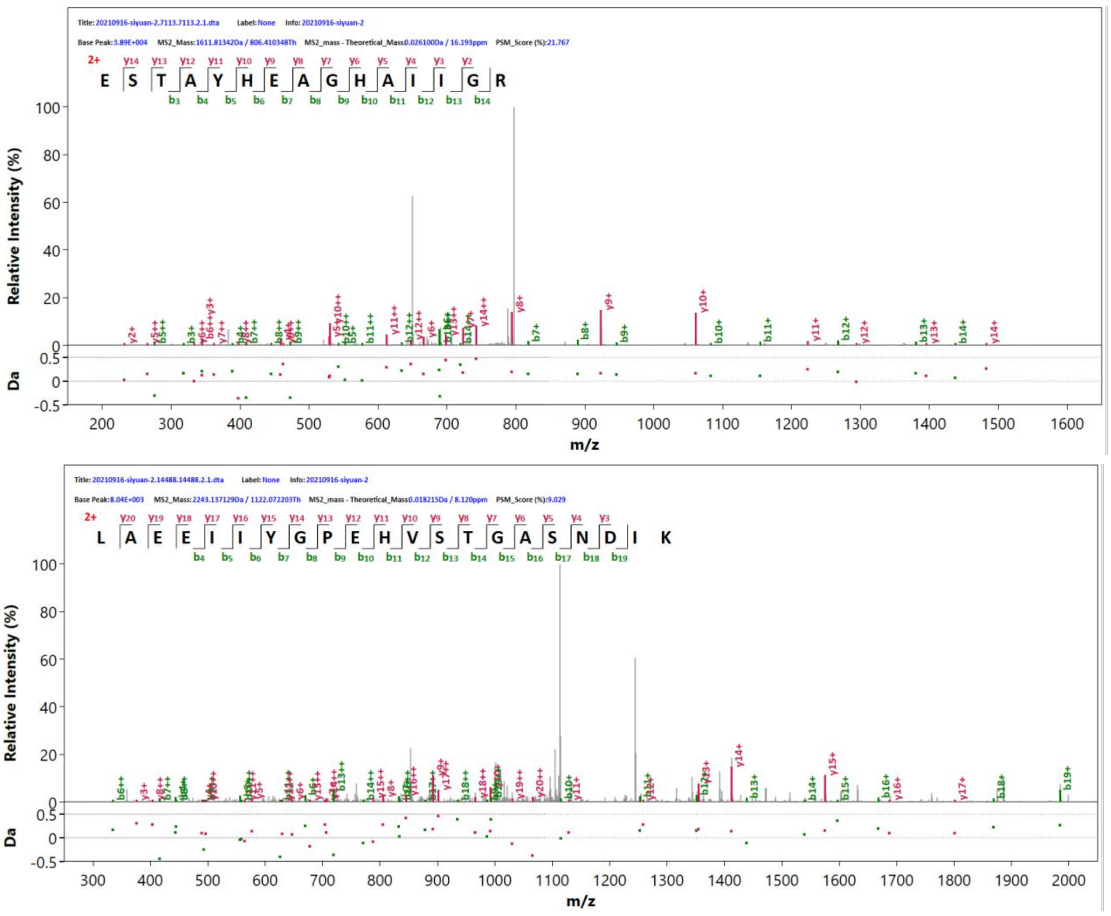

Figure S1 The differential peptides in ceftazidime resistance group.

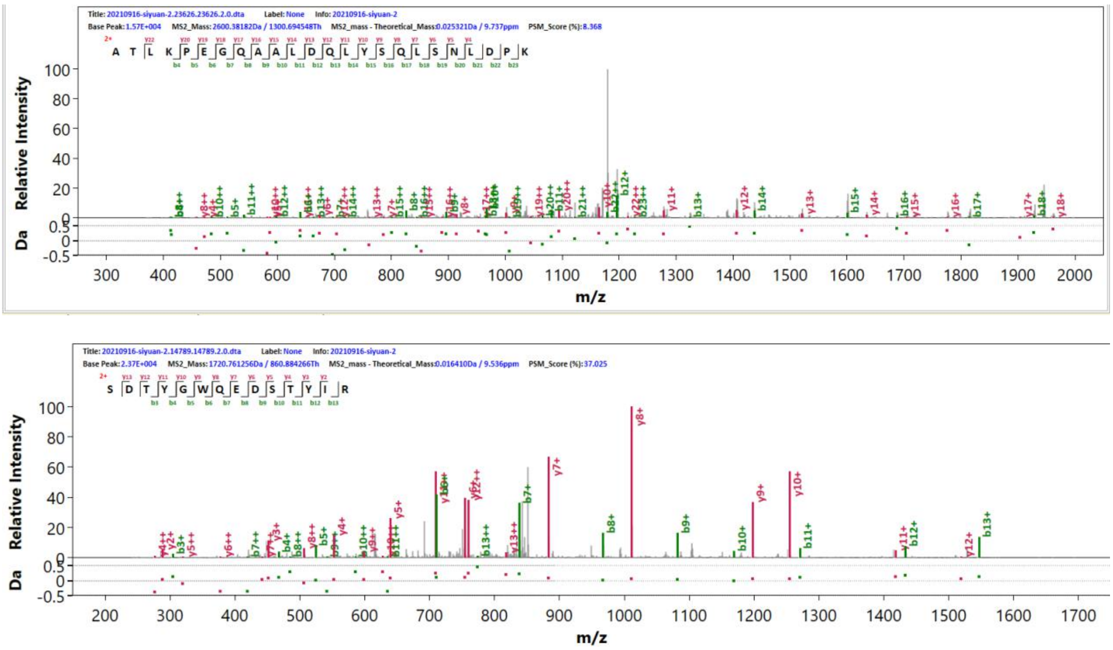

Figure S2 The differential peptides in piperacillin resistance group.

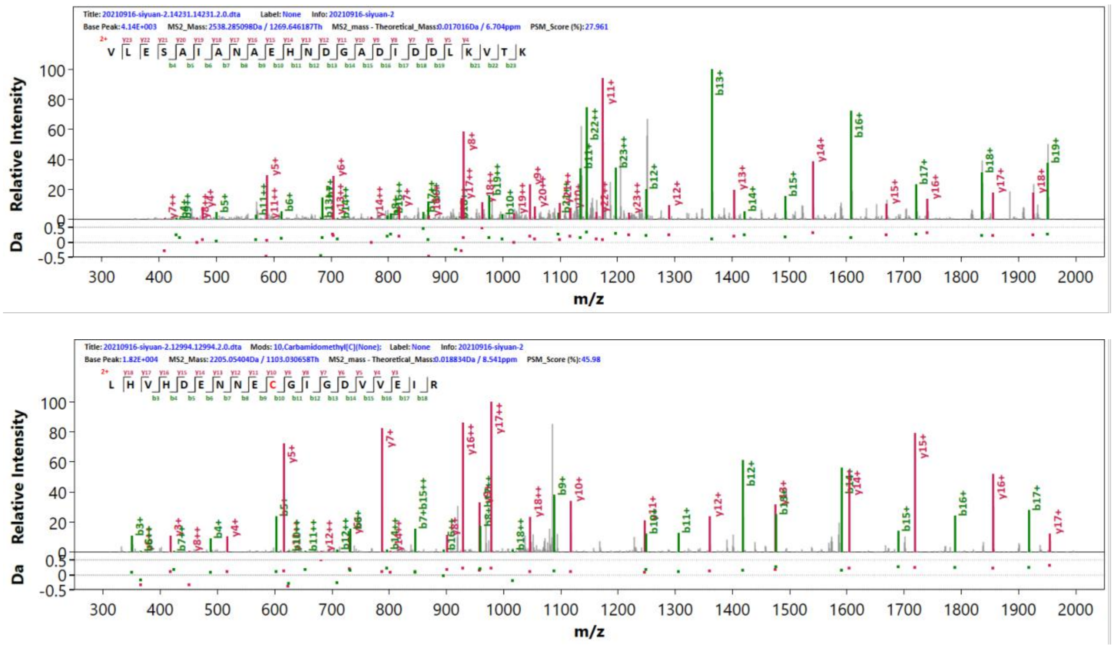

Figure S3 The differential peptides in levofloxacin resistance group.

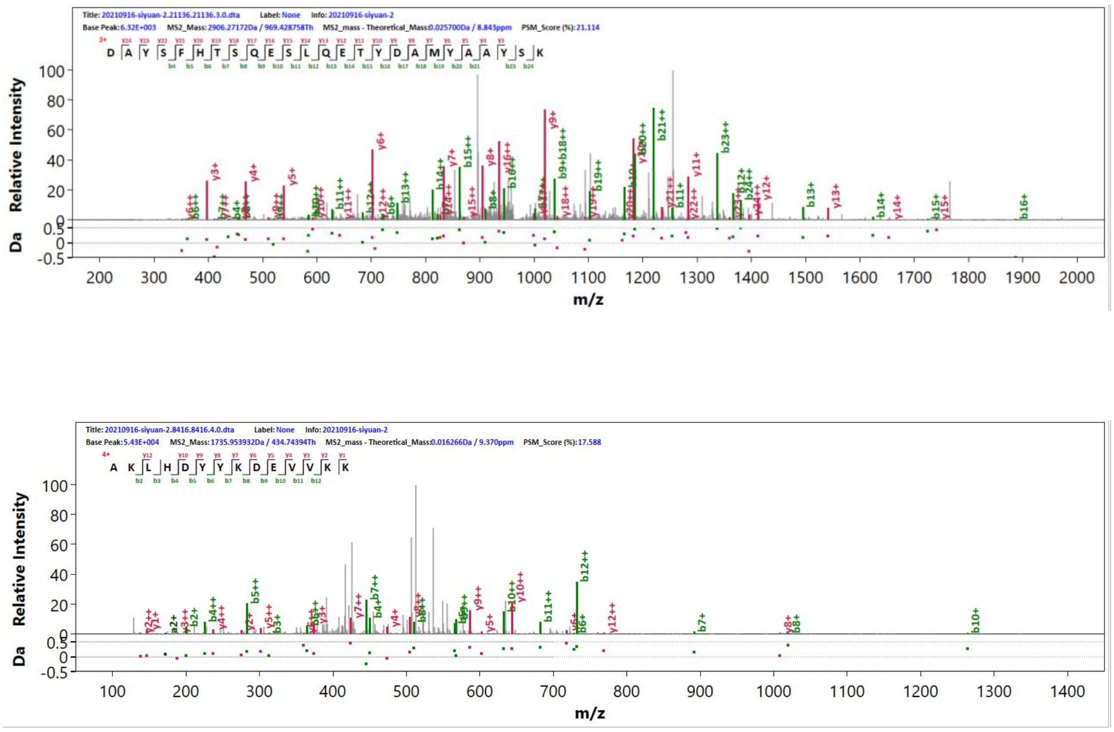

Figure S3 The differential peptides in tetracycline resistance group.

Table S1 The antimicrobial susceptibility of the Gram-negative strains isolated from DF patients

Bacterial species	Antibiotics	
	Ceftazidime	Piperacillin
Escherichia coli	R	R
Escherichia coli	R	R
Escherichia coli	R	R
Escherichia coli	S	S
Escherichia coli	S	R
Klebsiella pneumoniae	S	S
Klebsiella pneumoniae	S	R
Proteus penneri	S	
Enterobacter cloacae	R	S
Morganella morganii	S	S
Pseudomonas aeruginosa	S	S

S:Susceptibility; R:Resistance;I:intermediary

Table S2 The antimicrobial susceptibility of the Gram-positive strains isolated from DF patients

Bacterial species	Antibiotics	
	Levofloxacin	Tetracycline
Staphylococcus aureus （MSSA）	I	S
Staphylococcus aureus （MRSA）	S	S
Enterococcus faecalis	S	R
Enterococcus faecalis	S	R
Staphylococcus lentus	R	R

S:Susceptibility; R:Resistance;I:intermediary

Table S3 Detection of marker peaks in different bacteria resistant samples

Types	Maker Peaks	Sample 1	Sample 2	Sample 3	Sample 4	Sample 5	Sample 6	Sample 7
Ceftazidime resistance of bacillus	EALEWGTTGAGLR	Y	N	Y	N	Y	Y	N
	ESTAYHEAGHAIIGR	Y	Y	Y	Y	N	N	N
	LAEEIIYGPEHVSTGASNDIK	N	N	Y	Y	N	N	Y
		Sample 1	Sample 2	Sample 3	Sample 4	Sample 5		
Levofloxacin resistance of staphylococcus aureus	QHVPVFVTDRMVGHK	N	N	Y	Y	Y		
	VLESAIANARHNDGADIDDLKVTK	Y	Y	N	Y	N		
	LHVHDENNECGIGDVVEIR	N	N	N	N	Y		

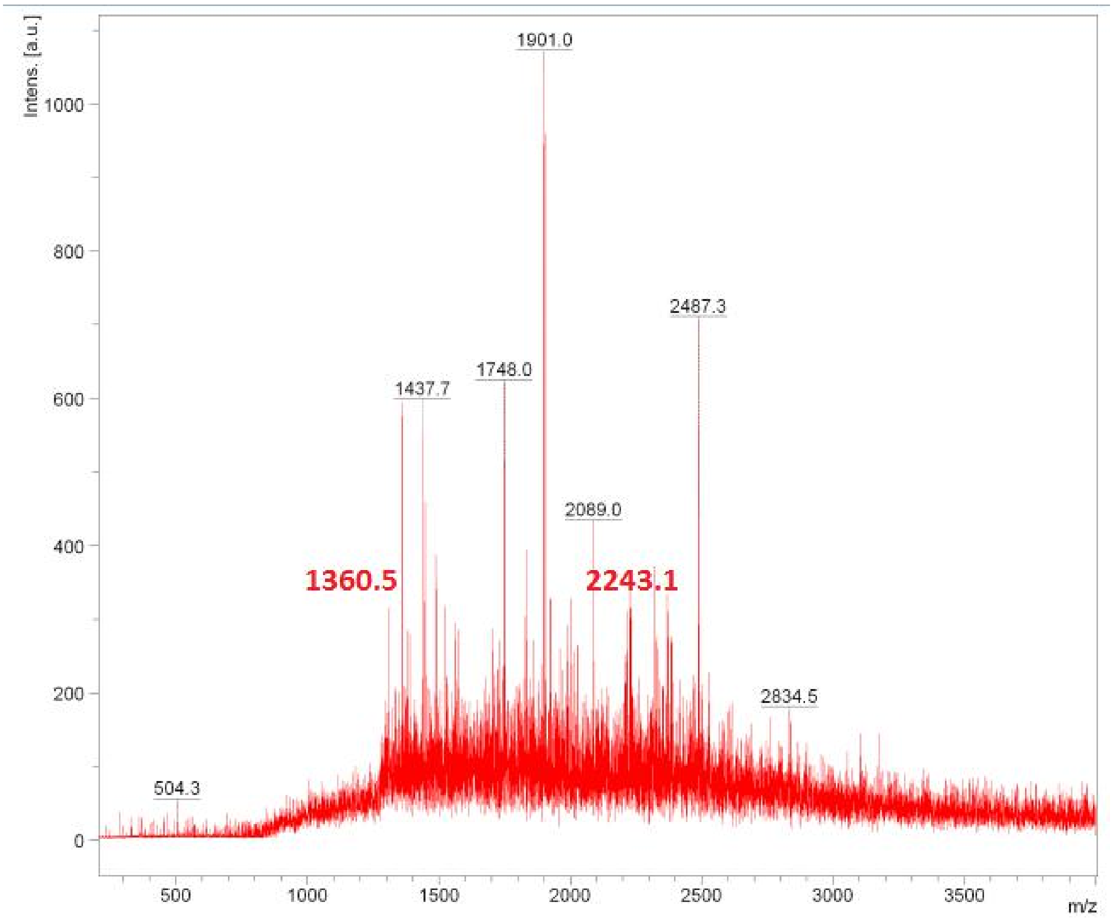

Figure S4 The detection of ceftazidime resistance of bacillus based on MALDI-TOF

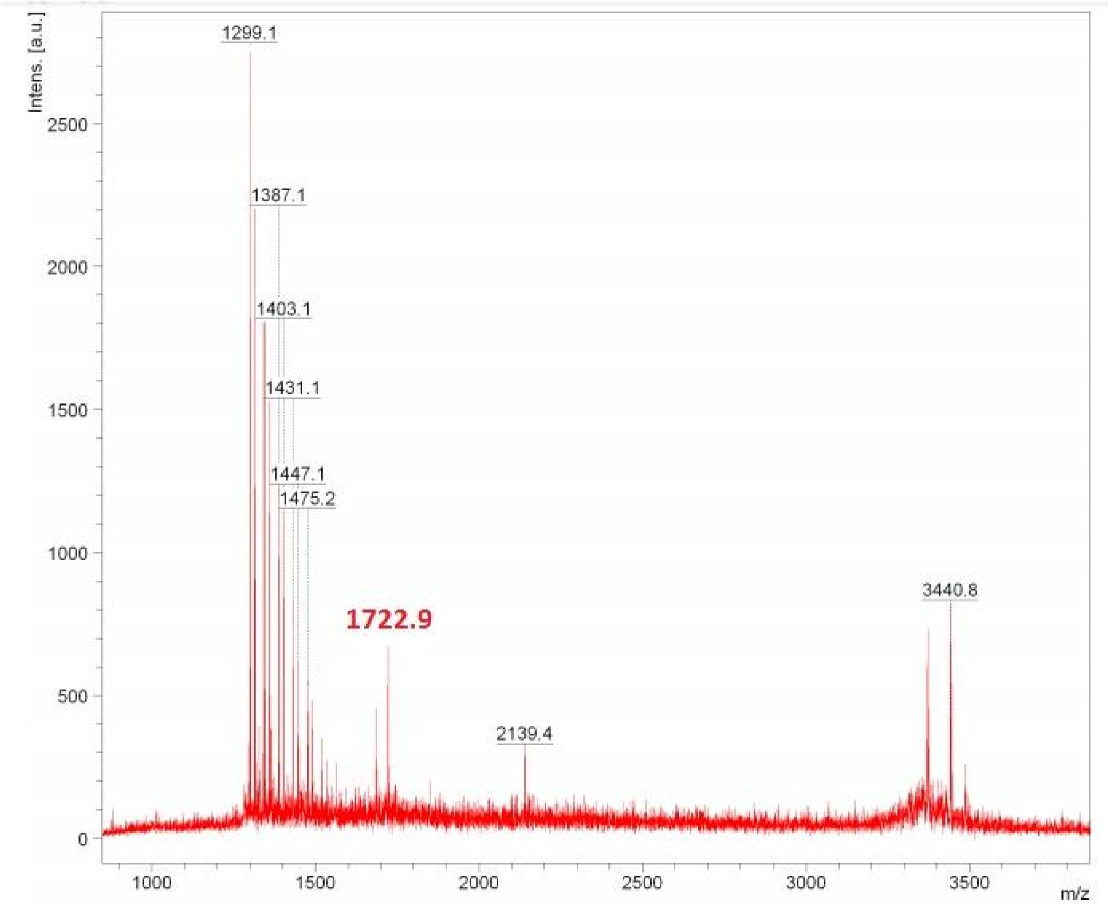

Figure S5 The detection of levofloxacin resistance of staphylococcus aureus based on MALDI-TOF
